# Application of SMILES-based molecular generative model in new drug design

**DOI:** 10.3389/fphar.2022.1046524

**Published:** 2022-10-13

**Authors:** Weiya Kong, Yuejuan Hu, Jiao Zhang, Qiaoyin Tan

**Affiliations:** ^1^ School of Sports Medicine and Rehabilitation, Beijing Sport University, Beijing, China; ^2^ Nursing Department of Fenyang College of Shanxi Medical University, Fenyang, China; ^3^ Innovation and Entrepreneurship College of Hunan University of Finance and Economics, Changsha, China; ^4^ College of Teacher Education, Zhejiang Normal University, Jinhua, China

**Keywords:** SMILES-based, molecular generative model, generative model, new drug design, drug design

## 1 Introduction

**FIGURE 1:**
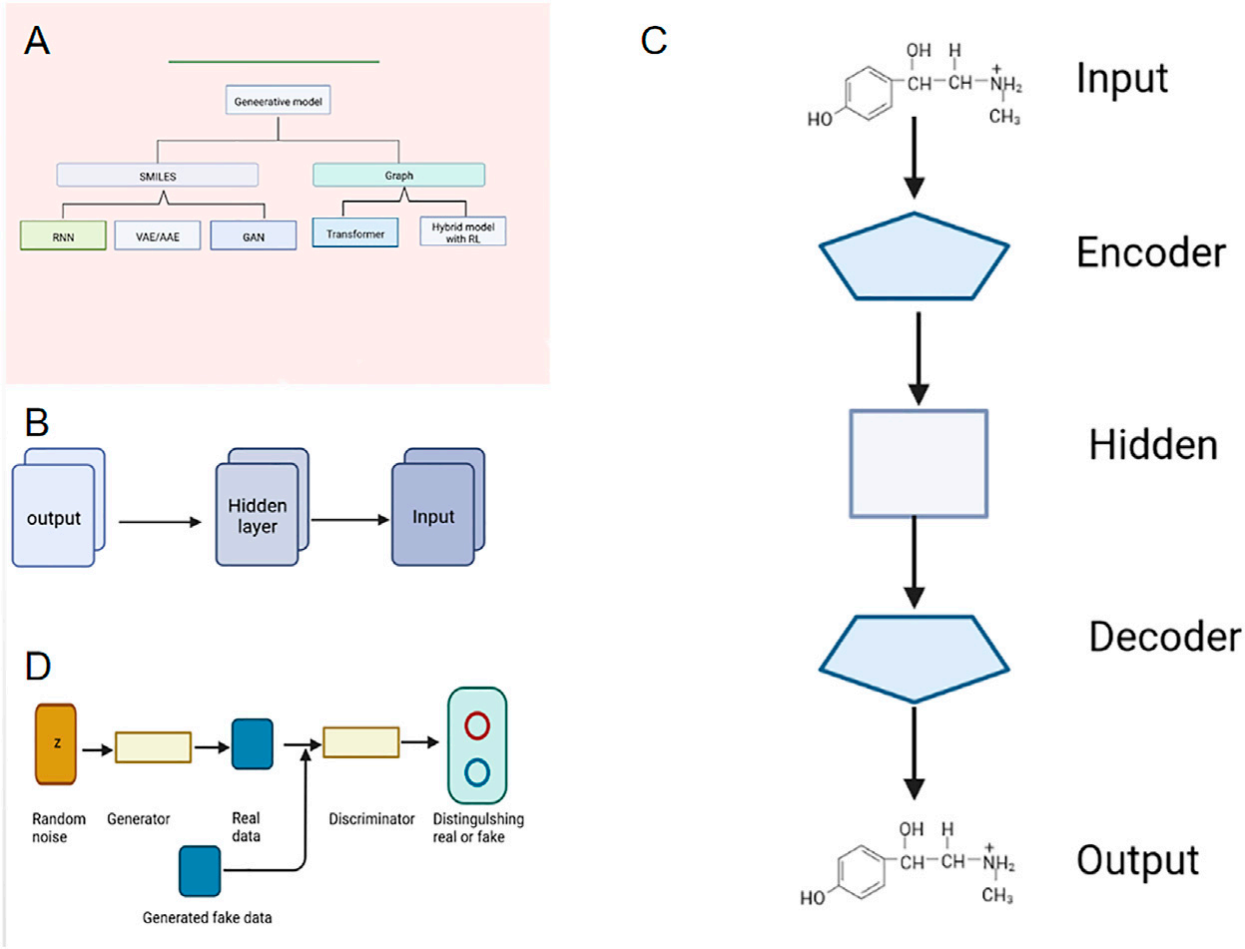
Different mode indication. **(A)** Generative Models for Molecular Design. **(B)** Structure of RNN. **(C)** Structure of VAE. **(D)** Structure of GAN.

Drugs are playing an increasingly important role in the long struggle between man and disease. Drug discovery is the process of identifying potential new therapeutic entities and drug design is the process of finding new medications based on knowledge of biological targets involving the design of molecules (Zhou and Zhong, 2017). Drug discovery and design has been facing obstacles due to the large human, material and financial resources required. With the success of artificial intelligence in the fields of image processing, pattern recognition and natural language processing (Xie et al., 2022), deep generative model has attracted wide attention in the field of drug discovery, and it also shows a promising application prospect in the field of molecular design optimization. When a generative model is used to generate molecules, its essence is to learn the distribution of molecules in the training set, and then generate molecules similar to but different from those in the training set. Combined with evolutionary algorithm or reinforcement learning, the properties of the generated molecules can be further optimized (Tong et al., 2021; Tan et al., 2022a). The molecular representation in the generative model can be in many forms, including Simplified Molecular Input Line Entry System (SMILES), molecular graph, etc. Generative models can be roughly divided into five categories, including recurrent neural network, RNN, autoencoder, AE, generative aggressive network, GAN, Transformer and generative model combined with reinforcement learning, RL (Bhisetti and Fang, 2022) as shown in Figure 1A. Among them, the molecular generative model based on the text sequence (SMILES) is the most widely used. This paper simply introduces the basic principle and application of deep generative model based on the latest molecular design of the text sequence (SMILES), so that readers can understand deep generative model and use it better in drug molecular design.

## 2 Model

### 2.1 RNN-based model

Recurrent neural network (RNN) can accept sequence data as input features. It was used in natural language processing, but now it is used to generate new compound structures (Wang et al., 2021). When RNN model is used to generate molecules, the molecules can be expressed as the sequence SMILES. Because SMILES is a string sequence, it is very suitable to be processed by a neural network like RNN, as shown in Figure 1B. It is found that the RNN-based method can learn the low-dimensional distribution of molecular sequence grammar and chemical space with the SMILES representation of compound molecules as input. RNN has unique advantages for sequences with large differences in length distribution (Cheng et al., 2021). Segler et al. (Segler et al., 2018) and Olivecrona et al. (Olivecrona et al., 2017) use RNN network with long short-term memory (LSTM) and gated recurrent unit (GRU) to generate molecular structures. By using a large number of SMILES molecular structures as training sets, the RNN model they trained can automatically generate molecular structures with high drug-like properties and the efficacy is as high as over 90%, and the diversity of molecular structures obtained is basically the same as that of the training set. RNN first learns how a large number of SMILES texts represent molecules in a language-like way, and the fitted model can generate new SMILES strings, i.e., new molecules without bias, which are suitable for virtual screening and other applications.

### 2.2 VAE-based model

VAE consists of encoder and decoder. The research group uses convolutional neural network (CNN) as an encoder to map the input molecular structure into latent variables, while the decoder uses RNN to recover the hidden variables to the SMILES sequence corresponding to the original molecular structure, as shown in Figure 1C . Due to the randomness of VAE algorithm, different hidden variables in the hidden variable space can be sampled after training, which can then be decoded to obtain different molecular structures (Altae-Tran et al., 2017; Wu et al., 2018). In addition, VAE can encode high-dimensional data in low-dimensional space, and form a “feature space” in parallel, which we can also call “drug space”. To some extent, it represents the complete set of targeted drugs. Therefore, if we take another point in this area and decode it back to the high-dimensional chemical molecule, then this molecule is a potential targeted new drug. However, VAE has limitations: if all the medicine-ready molecules are used to construct the “drug space”, then the medicine re-sampled is only a new medicine-ready molecule with no targeting selectivity; If the “drug space” is constructed with drugs targeting a certain pocket, a large number of known drugs targeting this site are needed, which cannot be used in the development of first-class drugs (Lim et al., 2018; Ragoza et al., 2022).

### 2.3 GAN-based model

The concept of GAN was proposed by Goodfellow in 2014, which was inspired by the zero-sum game. It is a type of neural network used in unsupervised learning, which helps to solve tasks such as generating images by text, improving the resolution of images, matching drugs, and retrieving images with specific patterns (Tong et al., 2021; Wang et al., 2021; Tan et al., 2022a; Bhisetti and Fang, 2022). The model consists of two neural networks: the generator G used to fit the data distribution and the discriminator D used to judge whether the input is “true”. In the training process, G outputs the hidden vector Z sampled from the prior distribution p(z) as a data space, while D distinguishes the real data from the output of the generated network as much as possible, thus forming a game process between the two networks to learn the production model of data distribution. Ideally, it will lead to a generative model that can be convincingly real (Kadurin et al., 2017), as shown in Figure 1D. In the molecular structure generation of GAN model, the generator generates random SMILES while the discriminator tries to distinguish these random SMILES from those of real molecules in the training set. In each round of training, the generator keeps learning, making the SMILES sequence generated by it closer and closer to the real molecule, until the discriminator cannot distinguish whether a SMILES sequence comes from the generator or the training set. Therefore, the generator network can be used to generate molecules (Polykovskiy et al., 2020).

## 3 Discussion

Some generative models have been successfully applied to generate new lead compounds with expected physical and chemical properties, but the application of generative models can be further explored in drug design.

Compared with the virtual compound library based on rules, the advantage of the generative model is that it can learn the joint probability distribution of molecular characterization and properties, which enables us to sample new molecules satisfying specific properties more effectively (Tong et al., 2021). Compared to the international chemical identifier (InCHI), which is also a one-dimensional linear representation, SMILES has a more rigorous syntax and uses a mapping algorithm from molecular graph to text (Elton et al., 2019). This makes SMILES easier to processing and more suitable for training machine learning models. The choice of SMILES as molecular input also does not suffer from the same limitations as fingerprints, i.e. the output is not directly converted into the true molecular structure and has difficulties in being used for *de novo* design. For generative models using 2D representations, i.e. molecular graph-based models, performance is often lacking in comparability due to different datasets and metrics; for generative models with 3D representations, they are limited to known molecular formulae only; whereas SMILES-based models are computationally lower cost, more easily scalable to larger molecules and/or larger datasets (Bilodeau et al., 2022), and can also benefit from improvements in algorithms related to natural language processing. Future research could translate specific target languages such as protein sequences into the SMILES language, i.e. the generation of molecules with specific characteristics could be considered as a translation. These methods may also be useful in bio drug design, such as stem cells (Tan et al., 2022b), growth factors (Tan et al., 2022c; Tan et al., 2022d), et al.

It is worth noting that despite the proliferation of SMILES-based models in recent years, it still has some limitations, such as the lack of explicit specification of molecular similarity, the possible inability to apply existing natural language processing models directly, and the need to additionally remove invalid SMILES. It is believed to be an important pillar in the field of new drug design in the near future, through continuous refinement to help pharmaceutical chemists expedite the process of drug discovery and design.
